# Potential Therapeutic Competition in Community-Living Older Adults in the U.S.: Use of Medications That May Adversely Affect a Coexisting Condition

**DOI:** 10.1371/journal.pone.0089447

**Published:** 2014-02-25

**Authors:** Songprod Jonathan Lorgunpai, Marianthe Grammas, David S. H. Lee, Gail McAvay, Peter Charpentier, Mary E. Tinetti

**Affiliations:** 1 Yale University School of Medicine, New Haven, Connecticut, United States of America; 2 Department of Medicine, Yale School of Medicine, New Haven, Connecticut, United States of America; 3 Oregon State University/Oregon Health and Science University, College of Pharmacy, Portland, Oregon, United States of America; 4 School of Epidemiology and Public Health, New Haven, Connecticut, United States of America; University of Glasgow, United Kingdom

## Abstract

**Objective:**

The 75% of older adults with multiple chronic conditions are at risk of therapeutic competition (i.e. treatment for one condition may adversely affect a coexisting condition). The objective was to determine the prevalence of potential therapeutic competition in community-living older adults.

**Methods:**

Cross-sectional descriptive study of a representative sample of 5,815 community-living adults 65 and older in the U.S, enrolled 2007–2009. The 14 most common chronic conditions treated with at least one medication were ascertained from Medicare claims. Medication classes recommended in national disease guidelines for these conditions and used by ≥2% of participants were identified from in-person interviews conducted 2008–2010. Criteria for potential therapeutic competition included: 1), well-acknowledged adverse medication effect; 2) mention in disease guidelines; or 3) report in a systematic review or two studies published since 2000. Outcomes included prevalence of situations of potential therapeutic competition and frequency of use of the medication in individuals with and without the competing condition.

**Results:**

Of 27 medication classes, 15 (55.5%) recommended for one study condition may adversely affect other study conditions. Among 91 possible pairs of study chronic conditions, 25 (27.5%) have at least one potential therapeutic competition. Among participants, 1,313 (22.6%) received at least one medication that may worsen a coexisting condition; 753 (13%) had multiple pairs of such competing conditions. For example, among 846 participants with hypertension and COPD, 16.2% used a nonselective beta-blocker. In only 6 of 37 cases (16.2%) of potential therapeutic competition were those with the competing condition less likely to receive the medication than those without the competing condition.

**Conclusions:**

One fifth of older Americans receive medications that may adversely affect coexisting conditions. Determining clinical outcomes in these situations is a research and clinical priority. Effects on coexisting conditions should be considered when prescribing medications.

## Introduction

Almost three quarters of older adults have multiple chronic conditions, also referred to as multi-morbidity.[1] The health care costs, adverse health effects, and treatment burden associated with multi-morbidity have been well chronicled.[2]–[8] Older adults with multi-morbidity are prescribed multiple medications for their individual conditions. While benefiting one condition, it is possible that some of these medications may adversely affect a coexisting condition, a situation we refer to as therapeutic competition. Therapeutic competition is one type of disease-drug interaction in which a treatment recommended for one condition may adversely affect (i.e. compete with) another coexisting condition.

A few well publicized cases of therapeutic competition, such as the effects of COX-2 inhibitors on arthritis versus heart disease or rosiglitazone on diabetes versus heart failure, have increased awareness of the potential adverse outcomes of therapeutic competition.[9]–[11] The extent of therapeutic competition remains unknown but may be widespread given the frequency of multi-morbidity in older adults and the emphasis of disease guidelines on prescribing one or more medications for treatment of chronic conditions. There has been no systematic examination of the prevalence of this problem.

In a nationally representative sample of older adults, we determined the prevalence of the most common pairs of coexisting chronic conditions in which a medication recommended by a national specialty organization for one condition may worsen the coexisting (i.e. competing) condition. Among all individuals with the chronic condition for which the medication is recommended, we compared the frequency of use of the medication in individuals with and without the competing condition.

## Methods

### Study Population and Data

Participants were members of the Medicare Current Beneficiary Survey. Medicare is the federal government health insurance program for essentially all persons aged 65 and older, and some younger people with disabilities, in the United States. The Medicare Current Beneficiary Survey is a nationally representative sample of Medicare beneficiaries obtained using stratified multi-stage sampling from the enrollment files of Centers for Medicare and Medicaid Services (CMS), the governmental agency that runs the Medicare program.[12], [13] A new cohort is added yearly; each cohort is then interviewed and followed for four years. The current study included cohort members enrolled from 2007–2009. Response rates for the baseline interview were 78.0%, 79.5%, and 77.5% for the 2007, 2008, and 2009 cohorts, respectively. For the current study, we included all cohort members who: 1) were age 65 years or older, 2) did not reside in a skilled nursing facility (medication data was not available for skilled nursing facility residents), 3) completed the in-person interview during which medications were ascertained, and 4) participated in the traditional fee-for-service Medicare. Only traditional Medicare beneficiaries were included because health claims used to ascertain chronic conditions were not available for the 25% of Medicare beneficiaries enrolled in a Health Maintenance Organization plan, referred to as Medicare Advantage. All 5,815 MCBS participants who met these inclusion criteria constituted the study population. The study was deemed exempt from review by the Yale University Human Investigation Committee because it involved existing, publically-available, de-identified data.

Socio-demographic, behavioral, and functional data were obtained from the Cost and Use files based on in-person interviews that occurred yearly; the baseline interview was used for the current study.[12] Dependency in basic activities of daily living (BADLs) was defined as not performing independently one or more of transferring, walking, dressing, bathing, eating or toileting. Medication use was ascertained from 2008–2010 Cost and Use files for cohort members.[12], [13] The data obtained during the in-person interviews are those included in Table 1. The Interviews were conducted by Westat Inc. under contract from CMS Further details on the interview process are available on the MCBS website.[12], [13]

**Table 1:** Characteristics of Participants by Number of Coexisting Chronic Condition Pairs (N = 5,815).

	No. of Pairs of Coexisting Chronic Conditions^a^			
	Total	0 Condition Pairs(N = 1273)	1–2 Condition Pairs (N = 763)	3+ Condition Pairs(N = 3779)
**Characteristics**	**No. (%)**			
Age ≥80 years	2000 (34.4)	313 (24.6)	225 (29.5)	1462 (38.7)
Female	3282 (56.4)	634 (49.8)	459 (60.2)	2189 (57.9)
Non-white	683 (11.8)	168 (13.2)	80 (10.5)	435 (11.5)
Less than high school graduate	1391 (24.0)	267 (21.0)	151 (19.8)	973 (25.8)
Income ≤ $25,000 per year	2647 (45.5)	550 (43.2)	316 (41.4)	1781 (47.1)
Dependent in any basic activity of daily living	1793 (31.0)	223 (17.6)	166 (21.8)	1404 (37.4)
Weight loss	200 (3.4)	17 (1.3)	14 (1.8)	169 (4.5)
Hospitalized in past year	1019 (17.5)	29 (2.3)	62 (8.1)	928 (24.6)
**Conditions**				
Hypertension	3976 (68.4)	144 (11.3)	462 (60.6)	3370 (89.2)
Hyperlipidemia	3467 (59.6)	84 (6.6)	360 (47.2)	3023 (80.0)
Osteoarthritis	2857 (49.1)	120 (9.4)	241 (31.6)	2496 (66.0)
Diabetes	1530 (26.3)	18 (1.4)	75 (9.8)	1437 (38.0)
Coronary artery disease	1469 (25.3)	11 (0.9)	43 (5.6)	1415 (37.4)
Gastroesophageal reflux disease/peptic ulcer disease	1213 (20.9)	25 (2.0)	44 (5.8)	1144 (30.3)
Chronic obstructive pulmonary disease	1014 (17.4)	19 (1.5)	55 (7.2)	940 (24.9)
Hypothyroidism	1027 (17.7)	12 (0.9)	67 (8.8)	948 (25.1)
Atrial Fibrillation	769 (13.2)	8 (0.6)	30 (3.9)	731 (19.3)
Heart Failure	666 (11.5)	3 (0.2)	8 (1.0)	655 (17.3)
Osteoporosis	636 (10.9)	5 (0.4)	55 (7.2)	576 (15.2)
Benign prostatic hypertrophy	619 (10.6)	24 (1.9)	49 (6.4)	546 (14.4)
Depression	442 (7.6)	5 (0.4)	20 (2.6)	417 (11.0)
Dementia	344 (5.9)	3 (0.2)	17 (2.2)	324 (8.6)
**Medications** b				
Statin	2658 (45.7)	318 (25.0)	285 (37.4)	2055 (54.4)
Angiotensin converting enzyme inhibitor or angiotensin receptor blocker	2559 (44.0)	321 (25.2)	262 (34.3)	1976 (52.3)
Beta-blocker^c^	2353 (40.5)	269 (21.1)	224 (29.4)	1860 (49.2)
Thiazides	2116 (36.4)	242 (19.0)	223 (29.2)	1651 (43.7)
Proton Pump inhibitor^d^	1490 (25.6)	167 (13.1)	110 (14.4)	1213 (32.1)
Calcium channel blocker^e^	1375 (23.6)	144 (11.3)	149 (19.5)	1082 (28.6)
Levothyroxine	1083 (18.6)	93 (7.3)	98 (12.8)	892 (23.6)
Corticosteroid	1012 (17.4)	122(9.6)	108 (14.2)	782 (20.7)
Bisphosphonate	702 (12.1)	99 (7.8)	98 (12.8)	505 (13.4)
Selective serotonin re-uptake inhibitor	676 (11.6)	64 (5.0)	56 (7.3)	556 (14.7)
Metformin	601 (10.3)	81 (6.4)	45 (5.9)	475 (12.6)
Warfarin	610 (10.5)	46 (3.6)	30 (3.9)	534 (14.1)
Beta agonist	586 (10.1)	68 (5.3)	43 (5.6)	475 (12.6)
Clopidogrel	560 (9.6)	35 (2.7)	33 (4.3)	492 (13.0)
Alpha-adrenergic blocker	486 (8.4)	76 (6.0)	49 (6.4)	361 (9.6)
Sulfonylurea	412 (7.1)	47 (3.7)	23 (3.0)	342 (9.1)
Insulin	314 (5.4)	25 (2.0)	14 (1.8)	275 (7.3)
Glitazone	225 (3.9)	27 (2.1)	15 (2.0)	183 (14.8)
Cox-2 inhibitor	196 (3.4)	15 (1.2)	36 (4.7)	145 (3.8)
Cholinesterase Inhibitor	256 (4.4)	23 (1.8)	20 (2.6)	213 (5.6)
Tricyclic Antidepressant	174 (3.0)	13 (1.0)	22 (2.9)	139 (3.7)
5-a-reductase inhibitor	244 (4.2)	47 (3.7)	16 (2.1)	181 (4.8)
Serotonin–norepinephrine reuptake inhibitor	155 (2.7)	10 (0.8)	14 (1.8)	131 (3.5)
Selective estrogen-receptor modulator	93 (1.6)	13 (1.0)	13 (1.7)	67 (1.8)

a The number of pairs of the 14 most common coexisting chronic conditions experienced by the MCBS cohort for which there is at least one prescription medication recommended by the national specialty organization for most individuals with the conditions. Those with zero condition pairs had only one of the 14 chronic conditions.

b Prescription medications given a GRADE A or B or equivalent level of recommendations by the national specialty organization guideline for one or more of the 14 chronic conditions. All medications recommended by a guideline are included if evidence grading not included in the guideline. Prescription medications used by at least 2% of study participants are included. For example, fibrates, nicotinic acid, and bile sequestrants are mentioned in guidelines for hyperlipidemia but the prevalence of use was low. Nonprescription medications (e.g. aspirin, nonsteroidal anti-inflammatory drugs, H_2_ receptor antagonists) were not available.

c Among the 2353 beta-blocker users, 1807 used a selective beta-blocker, 267 used a nonselective beta-blocker, and 279 used an alpha/beta-blocker.

d Proton pump inhibitors are likely underestimated because does not include over the counter.

e Among the 1375 calcium channel blocker users, 1142 used a dihydropyridine calcium channel blocker and 233 used a nondihydropyridine (primarily diltiazem; verapamil was used by <2% of the study population)

### Ascertainment of Study Chronic Conditions

Study conditions included all nonmalignant chronic conditions experienced by at least 5% of participants for which at least one oral or inhaled prescription medication is recommended by national disease guidelines for most persons with the condition. Chronic conditions were ascertained from hospital, outpatient, and physician claims data during the first two years of MCBS enrollment. At least one hospital or two nonhospital claims at least one month apart were required for every condition. All disease claims were assigned to a single level Clinical Classification System (CCS) code based on their International Classification of Diseases, 9th Revision, Clinical Modification (ICD-9 CM) codes.[14] When appropriate, clinically identical or similar disease codes were combined.

The chronic conditions meeting study criteria included atrial fibrillation, benign prostatic hypertrophy (BPH), coronary artery disease, chronic obstructive pulmonary disease (COPD), dementia, depression, diabetes (type 2), gastrointestinal esophageal reflux and peptic ulcer disease (GERD/PUD), heart failure, hyperlipidemia, hypertension, hypothyroidism, osteoarthritis, and osteoporosis. We determined the frequency of all pairs of these chronic conditions experienced by study participants.

### Ascertainment of Medications

Prescription medications were ascertained by direct observation of the medication containers of currently used medications during the year two in-home interviews which occurred between 2008–2010. Nonprescription medications were not available in the MCBS database. We categorized medications into medication classes based on the World Health Organization's Anatomical Therapeutic Chemical (ATC) classification system.[15]

### Identification of Condition Pairs with Potential Therapeutic Competition

Three investigators including two practicing geriatricians (MET, MG) and a PhD clinical pharmacologist and pharmacist (DSHL) reviewed the national disease guidelines for these 14 chronic conditions. Two investigators reviewed each guideline, identifying all medication classes that were recommended on a continual basis for most individuals with the condition. When there was more than one national U.S. specialty organization, we selected the most recent guideline published.[16]–[29] When the Grading of Recommendations, Assessment, Development and Evaluation (GRADE) system was used, all medication classes with an A (strong evidence) or B (moderate) grade were recorded.[30] When the GRADE system was not used, reviewers recorded medication classes with an evidence level of I or II. If no evidence grading was used, all medications recommended were recorded. For coronary artery disease (CAD), we identified medication recommendations for post myocardial infarction, acute coronary syndrome (ACS), and angina. Discrepancies among the medication lists generated by the three reviewers were reconciled by consensus. Medication subclasses (selective and nonselective beta-blockers and alpha-beta blockers and dihydropyridine and nondihydropyridine calcium channel blockers) were each considered separately when guidelines recommended for or against a medication subclass. For guidelines that did not stipulate subclass, we assumed all subclasses might be prescribed for the condition. Because it was not possible to determine for which of their coexisting conditions a guideline recommended medication was given, individuals were included in all possible potential therapeutic competition situations for which they had an indicated and competing condition and received a recommended medication for any indicated condition. For example, if an individual with diabetes received a glitazone and had both CAD and heart failure, that individual was included in potential therapeutic competition frequencies for both CAD and heart failure. The 27 prescription medication classes (including the three beta-blocker, and two calcium channel blocker, subclasses) meeting our selection criteria are listed in Table 1.

To determine which of the medication classes selected by review of the disease guidelines might constitute a possible therapeutic competition, we evaluated every combination of two coexisting conditions. For each combination of coexisting conditions, we first identified medications recommended for one of the conditions that are well acknowledged to adversely affect the coexisting condition (i.e. corticosteroids in persons with DM, osteoporosis, or GERD/PUD; warfarin in persons with GERD/PUD; tricyclic antidepressants in persons with CAD). For all other study medications, we considered a potential therapeutic competition to be present if: 1) adverse effects on a coexisting condition were mentioned in any of the national disease guidelines reviewed; [16]–[29] or 2) evidence of adverse effects on the coexisting condition was reported in a systematic review or at least two studies published since 2000. [9]–[11]; [31]–[63] To identify the most common situations of potential therapeutic competition, we limited this search to medications reported by at least 2% of participants.

### Statistical Analysis

Frequencies and percentages were calculated to describe the characteristics of the sample and the prevalence of chronic conditions and medication classes. Cross-sectional statistical weights, developed by Westat Inc. for MCBS, were used to estimate the number of persons in the U.S. population with potential therapeutic competition as represented by cohort members.[12], [13], [64] SAS version 9.3 software (SAS Institute Inc. 2011. SAS/STAT 9.3 User's Guide. Cary, NC) was used to compute risk differences and 95% confidence intervals between individuals with and without the competing conditions.

## Results

Thirty four percent of the 5,815 participants were age 80 years and over; 56.4% were women (Table 1). Hypertension (68.4%), hyperlipidemia (59.6%), and osteoarthritis (49.1%) were the most common chronic conditions. The most frequently reported prescription medications included angiotensin converting enzyme inhibitors or angiotensin receptor blockers (ACE/ARB) (44.0%) and statins (45.7%); 40.5% used beta-blockers. Among beta-blocker users, 76.8% received selective beta-blockers (Table 1). The prevalence of use of the 27 medication classes did not change from 2008 through 2010 except for an increase in statin use (42.4% in 2008; 46.5% in 2009; and 48.5% in 2010) and a decrease in bisphosphonate use (12.6% in 2008;13.8% in 2009; and 9.9% in 2010).

Among participants, 4542 (78.1%) suffered from at least one pair of coexisting study chronic conditions; 65.0% of participants had 3 or more pairs and 31.4% had at least 10 pairs of the study conditions. Increasing numbers of chronic condition pairs were associated with older age, greater dependencies in basic ADLs, and higher frequency of hospitalizations in the past year (Table 1).

### Prevalence of Potential Therapeutic Competition

Based on the criteria described in the Methods, 15 of the 27 medication classes (55.5%) recommended for one of the study conditions may adversely affect other study conditions. Among the 91 possible pairs of the study chronic conditions, 25 (27.5%) have at least one potential therapeutic competition. The prevalence of these chronic condition pairs and the frequency of use of medications that may adversely affect one or the other of the conditions are shown in Table 2. For example, among the 846 participants with coexisting hypertension and COPD, representing over 3.5 million older Americans, 16.2% used a nonselective beta-blocker or alpha/beta-blocker that might exacerbate their COPD while 39.6% received a beta agonist that could worsen their hypertension (Table 2). Among the estimated 1.2 million older adults with diabetes and heart failure, 27.3% received an alpha/beta-blocker that may cause orthostasis or syncope in those predisposed because of coexisting diabetic autonomic neuropathy; 10.3% used a glitazone that could exacerbate their heart failure.

**Table 2:** Prevalence of Potential Therapeutic Competition in Common Co-existing ChronicConditions among Community-living Persons in the U.S. Aged 65 and Older (N = 5815).

Competing Chronic Conditions^a^	No. Participants (%)	Population Estimates^b^	Competing Medication (%)^c^
Hypertension and osteoarthritis	2309 (39.7)	9,719,789	COX-2 inhibitor (5.3%)
Hypertension and diabetes	1384 (23.8)	6,086,828	Alpha/beta-blocker (11.4%)
Hypertension and Chronic obstructive pulmonary disease	846 (14.6)	3,812,031	Nonselective beta-blocker or alpha/beta-blocker (16.2%) Beta-agonists (39.6%) Corticosteroids (43.0%)
Diabetes and coronary artery disease d	601 (10.3)	2,538,530	Alpha/beta-blocker (19.1%)^e^ Sulfonylurea (23.3%) Glitazone (12.3%)
Coronary artery disease and Chronic obstructive pulmonary disease	433 (7.5)	1,706,201	Nonselective beta-blocker or alpha/beta-blocker (22.2%) Beta-agonists (37.0%)
Coronary artery disease and GERD/PUD	469 (8.1)	1,907,220	Clopidogrel (27.1%)
Hypertension and depression	370 (6.4)	1,695,472	Serotonin–norepinephrine reuptake inhibitor (15.4%)
Heart failure and osteoarthritis	421 (7.2)	1,568,261	COX-2 inhibitor (2.4%)
Chronic obstructive pulmonary disease and GERD/PUD	364 (6.3)	1,523,022	Corticosteroid (46.7%)
Diabetes and Chronic obstructive pulmonary disease	353 (6.1)	1,518,139	Corticosteroid (43.1%)
Diabetes and heart failure	300 (5.2)	1,182,354	Alpha/beta-blocker (27.3%) Glitazone (10.3%)
Heart failure and Chronic obstructive pulmonary disease	307 (5.3)	1,186,652	Nonselective beta-blocker or alpha/beta-blocker (29.6%)
Diabetes and atrial fibrillation	236 (4.1)	940,460	Alpha/beta-blocker (13.1%)
Diabetes and Benign prostatic hypertrophy	202 (3.5)	876,471	Alpha-adrenoreceptor antagonist (45.5%)
Chronic obstructive pulmonary disease and atrial fibrillation	225 (3.9)	875,313	Nonselective beta-blocker or alpha/beta-blocker (22.2%) Beta agonists (38.2%)
Osteoporosis and GERD/PUD	220 (3.8)	865,530	Proton pump inhibitor (64.1%)^f^ Bisphosphonate (40.9%)
Atrial fibrillation and GERD/PUD	217 (3.7)	829,600	Warfarin (53.9%) Clopidogrel (12.0%)
Coronary artery disease and depression	150 (2.6)	658,174	Tricyclic antidepressant (6.0%)
Diabetes and depression	142 (2.4)	645,978	Tricyclic antidepressant (6.3%)
Osteoporosis and Coronary artery disease	155 (2.7)	587,090	COX-2 inhibitor (3.2%)
Chronic obstructive pulmonary disease and osteoporosis	149 (2.6)	568,684	Corticosteroid (40.9%)
Diabetes and osteoporosis	127 (2.2)	517,475	Glitazone (12.6%)
Atrial fibrillation and osteoporosis	102 (1.8)	366,324	Bisphosphonate (40.2%)
Atrial fibrillation and depression	79 (1.4)	331,667	Tricyclic antidepressant (2.5%)
Atrial Fibrillation and dementia	79 (1.4)	279,358	Cholinesterase inhibitor (36.7%)

Abbreviations: GERD/PUD, gastroesophageal reflux disease or peptic ulcer disease.

a The most common pairs of coexisting chronic conditions listed in **Table 1** for which there is at least one medication (listed in **Table 1**) recommended by the national specialty organization guidelines that may worsen the other condition in the pair (see Methods for how these competing medications were identified). Of the guideline recommended medications, only prescription medications used by at least 2% of the study sample are included.

b Estimated number of persons in U.S. with the competing chronic conditions.

c Percent of participants with the coexisting conditions who use the potentially competing medication, that is, a medication recommended for one chronic condition that may worsen the coexisting chronic condition. See Methods for selection criteria for competing medications.

d Coronary artery disease includes history of myocardial infarction, acute coronary syndrome, or angina.

e Manifestations of diabetic autonomic neuropathy, such as orthostasis, may be worsened by alpha/beta-blockers.

f Proton pump inhibitors use is likely underestimated because nonprescription use of proton pump inhibitors not included in the dataset.

Among the 5,815 participants, 1,313 (22.6%) received at least one medication for a condition that may worsen a coexisting condition and therefore had at least one of the potential therapeutic competitions listed in Table 2. Of these individuals, 286 (4.9%) had two, while 468 (8.1%) had three or more pairs of coexisting conditions in which a medication received for one of their conditions may adversely affect the other condition.

### Medication Use in Participants with and without a Competing Condition

For each of the medications recommended by national disease guidelines for the 14 conditions, we determined whether the frequency of use differed by whether participants had a competing condition (Table 3). For only 6 of the 37 condition pairs (16.2%), were participants with the competing condition less likely (i.e. 95% CI for risk difference excluded 1) to receive the potentially offending medication than participants without the competing condition. For example, among individuals with atrial fibrillation, 5.8% of individuals with concomitant COPD used a nonselective beta-blocker versus 10.7% of those without COPD (risk difference −4.9; 95% CI −8.9,−0.9). For 67.6% of condition pairs (25/37), there was no difference in the frequency of use of a recommended medication between individuals who had, and those who did not have, the competing condition (Table 3). For five combinations of conditions (13.5%), participants who had a competing condition were more likely to receive the potentially harmful medication than participants without the competing condition (Table 3). For instance, 17.3% of individuals with coexisting atrial fibrillation and COPD received an alpha/beta-blocker that may exacerbate their COPD versus 9.4% of individuals with atrial fibrillation but no COPD (risk difference 8.0; 95% CI 2.4,13.5).

**Table 3:** Frequency of Use of a Recommended Medication for a Chronic Condition According to Presence of Competing Conditions among Community-living Persons in the U.S. Aged 65 and Older (N = 5815).

		Use of Recommended Medication when:		
Indicated Condition^a^	Competing Condition	Competing condition present^b^	Competing condition absent^c^	Risk Difference (95% Confidence interval)
		n/N (%)		
		**Alpha/Beta-blocker** d **[31.32]**		
Hypertension	Diabetes	158/1384 (11.4)	163/2592 (6.3)	5.1 (3.2, 7.1)
Hypertension	COPD	98/846 (11.6)	223/3130 (7.1)	4.5 (2.1, 6.8)
Coronary artery disease	Diabetes	115/601 (19.1)	97/868 (11.2)	8.0 (4.2, 11.7)
Coronary artery disease	COPD	78/433 (18.0)	134/1036 (12.9)	5.1 (0.9, 9.2)
Heart Failure	Diabetes	82/300 (27.3)	81/366 (22.1)	5.2 (−1.4, 11.8)
Heart Failure	COPD	82/307 (26.7)	81/359 (22.6)	4.1 (−2.4, 10.7)
Atrial fibrillation	Diabetes	31/236 (13.1)	59/533 (11.1)	2.1 (−3.0, 7.1)
Atrial fibrillation	COPD	39/225 (17.3)	51/544 (9.4)	8.0 (2.4, 13.5)
		**Nonselective Beta-blocker** d **[33.34]**		
Hypertension	COPD	41/846 (4.9)	207/3130 (6.6)	−1.8 (−3.5, −0.1)
Coronary artery disease	COPD	19/433 (4.4)	68/1036 (6.6)	−2.2 (−4.6, 0.3)
Atrial Fibrillation	COPD	13/225 (5.8)	58/544 (10.7)	−4.9 (−8.9, −0.9)
		**Beta-agonist** d [**35]**–**[37**]		
COPD	Hypertension	335/846 (39.6)	72/168 (42.9)	−3.3 (−11.4, 4.9)
COPD	CAD	160/433 (37.0)	247/581 (42.5)	−5.6 (−11.6, 0.5)
COPD	Atrial Fibrillation	86/225 (38.2)	321/789 (40.7)	−2.5 (−9.7, 4.8)
		**Corticosteroid**		
COPD	Hypertension	364/846 (43.0)	83/168 (49.0)	−6.4 (−14.6, 1.9)
COPD	Diabetes	152/353 (43.1)	295/661 (44.6)	−1.6 (−8.0, 4.8)
COPD	GERD/PUD	170/364 (46.7)	277/650 (42.6)	4.1 (−2.3, 10.5)
COPD	Osteoporosis	61/149 (40.9)	386/865 (44.6)	−3.7 (−12.3, 4.9)
		**Cox-2 inhibitor** d [**9]**, **[44]**–**[46**]		
Osteoarthritis	Hypertension	123/2309 (5.3)	34/548 (6.2)	−0.9 (−3.1, 1.3)
Osteoarthritis	CAD	37/875 (4.2)	120/1982 (6.0)	−1.8 (−3.5, −0.1)
Osteoarthritis	Heart failure	10/421 (2.4)	147/2436 (6.0)	−3.7 (−5.4, −1.9)
		**Sulfonylurea** d **[51.52]**		
Diabetes	Coronary artery disease	140/601 (23.3)	214/929 (23.0)	0.3 (−4.1, 4.6)
		**Glitazone** d [**10]**, **[47]**–**[50**]		
Diabetes	Coronary artery disease	74/601 (12.3)	118/929 (12.7)	−0.4 (−3.8, 3.0)
Diabetes	Heart failure	31/300 (10.3)	161/1230 (13.1)	−2.8 (−6.7, 1.2)
Diabetes	Osteoporosis	16/127 (12.6)	176/1403 (12.5)	0.1 (−6.0, 6.1)
		**Clopidogrel** d [**42]**, **[43**]		
Atrial fibrillation	GERD/PUD	26/217 (12.0)	66/552 (12.0)	0.0 (−5.1, 5.1)
Coronary artery disease	GERD/PUD	127/469 (27.1)	261/1000 (26.1)	1.0 (−3.9, 5.8)
		**Warfarin**		
Atrial fibrillation	GERD/PUD	117/217 (53.9)	322/552 (58.3)	−4.4 (−12.2, 3.4)
		**Serotonin norepinephrine reuptake inhibitor** d [**56]**, **[57**]		
Depression	Hypertension	57/370 (15.4)	13/72 (18.1)	−2.7 (−12.3, 7.0)
		**Tricyclic antidepressant** d		
Depression	Atrial fibrillation	2/79 (2.5)	28/363 (7.7)	−5.2 (−9.6, −0.8)
Depression	Coronary artery disease	9/150 (6.0)	21/292 (7.2)	−1.2 (−6.0, 3.6)
Depression	Diabetes	9/142 (6.3)	21/300 (7.0)	−0.7 (−5.6, 4.3)
		**Cholinesterase inhibitor** d [**59]**–**[61**]		
Dementia	Atrial fibrillation	29/79 (36.7)	141/265 (53.2)	−16.5 (−28.7, −4.3)
		**Alpha-adrenergic antagonist** d [**26]**, **[62]**, **[63**]		
Benign prostatic hypertrophy	Diabetes	92/202 (45.5)	156/417 (37.4)	8.1 (−0.2, 16.4)
		**Proton pump inhibitor** d [53]–[55]		
GERD/PUD	Osteoporosis	141/220 (64.1)	598/993 (60.2)	3.9 (−3.2, 10.9)
		**Bisphosphonate** d [**38]**–**[41**]		
Osteoporosis	GERD/PUD	90/220 (40.9)	208/416 (50.0)	−9.1 (−17.2, −1.0)
Osteoporosis	Atrial fibrillation	41/102 (40.2)	257/534 (48.1)	−7.9 (−18.4, 2.5)

Abbreviations: COPD, chronic obstructive pulmonary disease; GERD/PUD, gastroesophageal reflux disease or peptic ulcer disease;

a The first chronic condition listed for a pair is the condition for which the medication is recommended by the national specialty organization guideline; the second chronic condition in a pair is the coexisting condition that may be worsened with the medication (i.e. competing condition).

b The numerator is the number of participants with the indicated condition who received the recommended medication. The denominator is the number of participants who had the indicated condition who also had the competing condition.

c The numerator is the number of participants with the indicated condition who received the recommended medication. The denominator is the number of participants who had the indicated condition but did not have the competing condition.

d Numbers in brackets are the reference of the studies supporting the possible adverse effect of the medication class on the competing condition.

## Discussion

In this nationally representative sample of older adults in the U.S., over 20% took at least one medication that could adversely affect another of their chronic conditions. Because MCBS is a nationally representative sample, study estimates reflect the prevalence of potential therapeutic competition in the older U.S. population. The frequency of potential therapeutic competition is likely related to the high prevalence of multi-morbidity in older adults combined with the focus of disease guidelines on medication benefits for individual conditions.

A few medications such as non-selective beta blockers, Cox 2 inhibitors, and bisphosphonates were used less frequently in those with, than without, a competing condition suggesting that clinicians did consider adverse effects on coexisting conditions in their clinical decision-making. In many cases, however, the medications were used at least as often in those who had the competing condition than in those who did not. The aim of this study was to identify situations of potential therapeutic competition and estimate the frequency of such situations in older adults. It remains to be determined how frequently adverse clinical outcomes occur in these situations.

Recent studies in other developed countries report similar rates of use of study medication classes as in the U.S.[65], [66] Renin angiotensin system medications, for example, were used by 44%, 26%, and 32% of community-living older adults in the U.S., Sweden, and Finland respectively. The comparable percentages were 40%, 33%, and 53% for beta-blockers, and 24%, 17%, and 23% for calcium channel blockers. These comparisons suggest that potential therapeutic competition may be a common concern across developed countries with growing populations of older adults with multiple chronic conditions.

Because Medicare HMO (Medicare Advantage) patients are healthier than their age-matched traditional Medicare beneficiaries, their exclusion may have resulted in overestimating the prevalence of potential therapeutic competition. Although there is no gold standard for determining what medications community-living older adults actually take, the direct observation of the medications in the home has been shown to be more accurate and reliable than other methods such as medication interview, medication lists, or “brown bag” in the clinic.[67] For medications used for multiple conditions, we could not be sure for which condition a medication was prescribed although we do know that participants had the study condition and received the medication. Because the data were unavailable, we were unable to assess the prevalence of potential therapeutic competition for NSAIDS, aspirin, and other nonprescription medications. For conditions in which medication recommendations depend on type, severity, or stage (e.g. heart failure), we lacked the data to determine if individuals met criteria for this type or stage.

The prevalence of individual chronic conditions and combination of conditions vary depending on criteria for diagnosis and method of ascertainment.[2] The limitations of Medicare claims data for ascertaining chronic conditions have been well-chronicled with conditions that provide more lucrative reimbursement and require more frequent medical attention being more thoroughly reported.[6], [68], [69] We used two years of inpatient and outpatient claims to ascertain chronic conditions, thus increasing the likelihood of ascertainment. The prevalence for all of the conditions except dementia and depression were similar to those reported on the Center for Medicare and Medicaid's ’ Chronic Conditions Dashboard.[70] The underestimate of dementia and depression in claims data has been reported in previous studies.[71] The underreporting of some conditions suggests that we may have underestimated the frequency of some pairs of competing conditions. However, as we matched medications to chronic conditions, the likelihood is high that the condition was present when there were claims.

The medications were ascertained from 2008–2010; patterns of use may have changed for some medications since then although we detected few changes over the three study years. Other than subclasses of beta-blockers and calcium channel blockers, we combined all medications within a class; effects may vary within a class. Furthermore, the effects of medications vary by route. The effect of oral glucocorticoids on osteoporosis or PUD, for instance, is different than the effect of inhaled glucocorticoids.

Some of the therapeutic competitions included in this study, such as warfarin in individuals with atrial fibrillation and PUD or glitazones in individuals with diabetes and heart failure, are well established. Determining from the available evidence whether the other medications qualified for possible therapeutic competition was challenging. There is no standard for determining harm of medications that parallels attempts to ascertain benefits. Any approach, therefore, will have limitations. An inherent problem is that adverse effects are not as carefully assessed as benefits. Clinical trials focus on evaluating benefit of medications on the indicated disease in relatively homogenous populations of younger populations with fewer chronic conditions than clinical populations of older adults. RCTs thus likely underestimate the frequency of adverse effects in clinical populations. On the other hand, observational studies, while providing evidence from actual clinical practice, are prone to bias. We attempted to be systematic and limited the current report to medications with at least two studies showing an adverse effect on the competing conditions. For some medications, the evidence remains conflicting across studies. For many of the medications that met criteria for inclusion, there were also reports that did not suggest harm. The same is true for well accepted evidence of benefits for many medications. We included medications if the preponderance of evidence supports potential therapeutic competition, such as nonselective β-Blockers with COPD. However, we did not include situations which are more uncertain such as β-Blockers with depression, nondihydropyridine CCBs in heart failure, or statins with dementia.[72] Unfortunately, because adverse consequences of medications have not been consistently measured, it currently is not possible to assess the strength of the evidence for harm in the way benefits are assessed.

The list of potentially competing conditions reported in this study is not exhaustive. To focus on the most common clinical situations, we investigated only medications used by at least 2% of participants and chronic conditions with a prevalence of at least 5%. To introduce the concept of therapeutic competition, we limited the study to pairs of coexisting conditions. Patients with MCC, however, have combinations of three, four, and more chronic conditions. Eventually, the effect of treatments for various combinations of conditions will need to be explored.

While we studied one potential mechanism of adverse medication effect in older adults with multi-morbidity, medications can adversely affect individuals through several other mechanisms. For example, many medications contribute to geriatric syndromes such as falls and delirium. Chronic kidney disease can exacerbate the adverse consequences of several medications. Treatment for one condition may mask the adverse effects of treatment of another condition such as β-blockers masking the hypoglycemic effects of anti-diabetic agents. Furthermore, medications themselves cause adverse effects such as dizziness, fatigue, and anorexia in older adults.[73]

The implications of our findings are several. We quantified the magnitude of these tradeoff decisions that face clinicians, although we cannot comment on the appropriateness of these decisions. It is likely that many of the individuals experienced net benefit from the medications despite the presence of a competing condition. The presence of competing conditions does not imply contraindication of the medication but rather the need for clinicians to weigh the effects of medications on each of a patient's conditions, not just the condition for which it is recommended. Unfortunately, such evidence is lacking currently for many medications and chronic conditions. Studies of medication effects should include equally rigorous ascertainment of harms as well as benefits, not just on the disease of interest but on commonly co-existing conditions.

Evaluating the benefits and harms of cross-disease treatment regimens in individuals with common combinations of chronic conditions should be a focus of comparative effectiveness research as should identification of effective treatments that circumvent therapeutic competition. The current approach of adding a medication, such as adding a PPI to clopidogrel or corticosteroids to reduce the risk of gastrointestinal bleeding in those with PUD, may unintentionally substitute one therapeutic competition for another while adding to polypharmacy.

Currently, few guidelines developed by national specialty organizations address the harms and benefits of recommended medications in individuals with competing conditions or consider co-occurring conditions when making treatment recommendations. Recent reports suggest how guidelines could be adapted and presented in formats more useful for decision-making for patients with multi-morbidity.[74], [75] At the least, guideline developers should consider how commonly coexisting conditions should influence medication recommendations.[74], [75] Eventually, guideline developers and clinicians hopefully will be able to recommend medications based on evidence of absolute benefit versus harm for cross-disease universal health outcomes that are of greatest priority to individual patients such as survival, symptom burden, and function.[76]

Given the large number of potential therapeutic competitions, an evidence-based rating system that weighs the net benefit or harm of medications in persons with the coexisting conditions would help aggregate and prioritize the large amount of information for use in decision-making. The approach used to develop the Beers Criteria might serve as a model for evaluating and translating the evidence into clinically useful guidelines.[77] These evidence-based guidelines could also inform development of quality indicators of appropriate prescribing for patients with multiple chronic conditions.[78] Electronic health records, which currently check for only interactions among medications, should also include a check for interactions between medications and coexisting competing conditions.

One fifth of older adults are prescribed a medication that may adversely affect a coexisting condition. Determining the likelihood of net benefit or harm in these situations is a research and clinical priority. In addition to considering the effect of medications on coexisting conditions, heightened awareness of therapeutic competition should trigger systematic attention to identifying strategies for avoiding poor clinical outcomes in individuals with competing chronic conditions.
